# Native myocardial T1 precision is increased by correcting for myocardial blood variation

**DOI:** 10.1186/1532-429X-17-S1-Q47

**Published:** 2015-02-03

**Authors:** Jannike Nickander, Magnus Lundin, Goran Abdula, Peder Sörensson, James Moon, Peter Kellman, Andreas Sigfridsson, Martin Ugander

**Affiliations:** 1 Karolinska Institutet, Stockholm, Sweden; 2 Institute of Cardiovascular Science, London, UK; 3 National Institutes of Health, Bethesda, MD, USA

## Background

Native myocardial T1 mapping with CMR has emerged as a new diagnostic tool in heart disease. However, myocardial T1 will be affected by the T1 of blood present in the myocardium. To explore this, as there is no easy way to manipulate blood in isolation, we hypothesized that the population standard deviation (SD) for myocardial T1 could be reduced by correcting the myocardial T1 for characteristics of the blood. We sought to compare methods for doing this.

## Methods

Consecutive patients (n=100, age 53+/-17 years, 74 % male) underwent clinical CMR at 1.5T (Siemens Aera). A modified Look-Locker inversion recovery (MOLLI) sequence was used to acquire T1 and T1* maps. Native myocardial T1 values from the midmural ventricular septum were measured in a mid-ventricular short-axis T1 map. Hematocrit was acquired by venous blood sampling. T1 and T1* values of blood were measured in the blood pool of the left and right ventricle (LV and RV). Myocardial T1 was corrected according to the formula T1corrected = T1uncorrected + slope (mean(X)-X), where X is the blood measurement of T1, T1* or hematocrit, and the slope was calculated as the slope of the linear regression between myocardial T1 and the respective measure.

## Results

The hematocrit range was 34 to 52%. Mean uncorrected native myocardial T1 was 1021ms with a SD of 42ms. LV and RV blood T1, T1* and hematocrit correlated with myocardial T1 (R^2^ range 0.16 to 0.29). Using the blood parameters, the best model (consisting of LV blood T1 and T1*) could reduce the population myocardial T1 variation by 18% (SD 41.9ms to 34.3ms), see figure.

**Figure 1:**
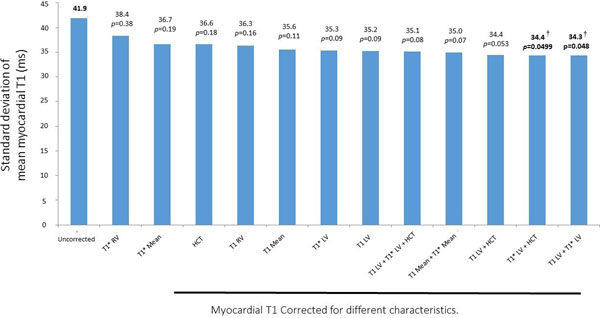


## Conclusions

Correcting native myocardial T1 for variations in blood parameters may reduce the normal variation and hence increase disease detection. The optimal model here was to correct for T1 and T1* of the LV blood pool, providing an 18% reduction in SD of native myocardial T1 measurement in a population. Correction for hematocrit did not provide an incremental improvement in precision. Image-based correction for blood T1 and/or T1* is feasible for clinical use and may improve disease detection in the clinical evaluation of native myocardial T1.

## Funding

The research was funded in part by the Swedish Research Council, Swedish Heart and Lung Foundation and the Stockholm County Council. Karolinska Institute has a research and development agreement regarding CMR with Siemens.

